# Improvement of Hip Pain After Total Hip Arthroplasty With Dry Needling as an Adjunct to Conventional Physiotherapy: A Case Series

**DOI:** 10.7759/cureus.32098

**Published:** 2022-12-01

**Authors:** Anthony Baumann, Robert J Trager

**Affiliations:** 1 Department of Rehabilitation Services, University Hospitals Cleveland Medical Center, Cleveland, USA; 2 Department of Chiropractic, University Hospitals Cleveland Medical Center, Cleveland, USA

**Keywords:** hip, rehabilitation, physical therapy, dry needling, total hip arthroplasty

## Abstract

While total hip arthroplasty (THA) is one of the most common and successful orthopedic surgeries, some patients may experience persistent, recurrent, or new hip pain despite successful THA. Dry needling (DN) is a common treatment for musculoskeletal pain, yet little data has been published on the use of DN on hip pain after THA. This series highlights two patients with prior THA and current hip pain that improved with DN used alongside conventional physiotherapy exercises.

Patient 1, a 70-year-old male four years post left THA, presented to a physical therapist with a three-year history of left hip pain. Patient 2, a 65-year-old female 10 years post right THA, presented with a one-month history of right hip pain after a fall. Both patients were reported to have a stable prosthesis without clinical or radiological evidence of loosening or other major complications. Examination of both patients revealed decreased hip range of motion, decreased hip strength, and lateral hip trigger points suggestive of a muscular origin of pain. The physical therapist treated both patients with DN alongside strengthening and stretching exercises, yielding significant improvements in pain severity, function, and range of motion.

These cases illustrate the successful use of DN alongside conventional physiotherapy to alleviate hip pain in patients with previous THA. Further research is needed to examine the efficacy and safety of DN for hip pain in individuals with prior THA.

## Introduction

Total hip arthroplasty (THA) is one of the most common and successful orthopedic surgical procedures, often resulting in improved pain and function [1]. However, a minority of patients who undergo successful THA report chronic postoperative pain or experience a new type of hip pain months or years following surgery [2]. Although sources of hip pain after THA may be frequently under-recognized, they may be treatable using conservative measures [2].

According to one survey study, about 28% of patients reported hip pain 12-18 months following a successful THA [3]. While persistent symptoms immediately following THA can be related to aseptic loosening, infection, or other serious etiologies, recurrent or new symptoms after a technically satisfactory THA are often musculoskeletal in origin [2]. Common causes of recurrent or new hip pain after THA include tendinitis and greater trochanteric pain syndrome, including abductor tendinopathy and snapping hip syndrome [2].

While hip pain after THA is relatively common, few studies have investigated or described a management algorithm for this condition [2,4]. Diagnosis of the pain source is often based on clinical findings and may be supported by other testing methods such as diagnostic ultrasound or magnetic resonance imaging [2,4]. Treatments for hip pain after THA may include non-steroidal anti-inflammatory medications, steroid and anesthetic injections, rest, and physical therapy depending on the source of symptoms [1,2].

Dry needling (DN) is a common conservative treatment used by physical therapists, chiropractors, and other practitioners to treat musculoskeletal pain, and is described as the insertion of thin monofilament needles into muscles, ligaments, tendons, and/or scar tissue [5]. DN has been shown to be effective for treating hip pain in those without prior THA [5], and emerging research suggests it may also be effective for treating chronic pain after total knee arthroplasty (TKA) [6]. However, based on a literature search of Google Scholar and PubMed on November 8, 2022, using search terms for “dry needling” and “total hip arthroplasty,” we were unable to find any literature describing the use of DN for patients with previous THA.

Given that hip pain after THA is common yet there is limited research describing its treatment, this case series highlights the effectiveness of adding DN to a standard physical therapy regimen when treating hip pain in patients with prior THA.

## Case presentation

Case 1

A 70-year-old man four years status post left THA with normal body mass index (BMI) with no other significant past medical history presented to the outpatient orthopedic physical therapy clinic in 2020 with a three-year history of left lateral hip and groin pain rated a mean 7/10 severity (numeric pain rating scale). The patient initially had left THA due to significant osteoarthritis in the left hip. The patient reported that he had seen multiple orthopedic surgeons who told him that nothing was structurally wrong with his hip prosthesis per radiograph imaging assessment. The patient also reported that he underwent conventional physical therapy at the time of symptom onset, which included left hip strengthening and iliopsoas stretching exercises, yet did not have significant relief from his symptoms. The patient suspected that he had “multiple trigger points” affecting his outer hip and thus presented to our physical therapy office to try DN. He reported being unable to play golf and was eager to return to playing. A Modified Oswestry Disability Index indicated a severe level of disability (56%).

On observation, the physical therapist observed that the patient ambulated with a mild Trendelenburg limp without an assistive device. Examination revealed a reduced passive range of motion of the left hip in all directions and reduced strength of 4-/5 to 4/5 in all tested actions (Table 1). The patient also demonstrated moderate tenderness to palpation at the left greater trochanter and had trigger points in the left gluteus minimus, gluteus medius, gluteus maximus, and piriformis. An examination of sensation and muscle stretch reflexes was normal. Due to the presence of tenderness to palpation at the lateral hip, gait abnormality, and lack of structural THA abnormality or signs of lumbar radicular syndrome to explain the patient’s symptoms, the physical therapist provided a working diagnosis of greater trochanteric pain syndrome. Differential diagnosis also included iliopsoas strain and adductor strain. Given the failure of the previous physical therapy regimen, the current treatments focused on lateral hip impairments and added DN as a therapy.

**Table 1:** Initial and follow-up examination and outcome measures for Case 1 Abbreviations: numeric pain rating scale (NPRS), Oswestry Disability Index (ODI) *Strength testing was scored out of a maximum of 5 (modified Medical Research Council manual muscle testing scale)

	Initial evaluation	Final follow-up
Passive hip range of motion (degrees)		
Flexion	90	110
Internal rotation	20	30
External rotation	40	50
Hip strength*		
Flexion	4	5
Abduction	4-	5
Internal rotation	4	5
External rotation	4	5
Disability measures		
Modified ODI raw score	56/100	6/100
Modified ODI disability (%)	56	6
Pain (NPRS)	7/10	0/10

**Figure 1:**
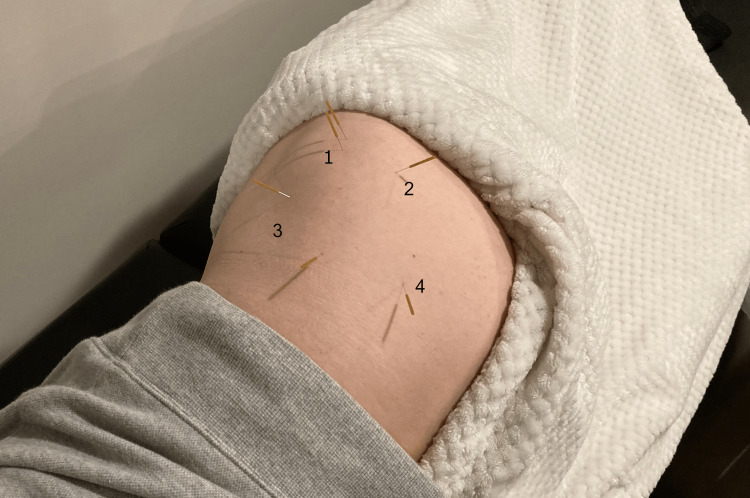
Lateral hip dry needling Dry needling locations for (1) trochanteric region, (2) piriformis, (3) gluteus minimus, (4) gluteus medius

The physical therapist recommended a treatment plan (two visits per week over one month) to which the patient consented, and at each visit performed DN targeting the greater trochanteric region and gluteal trigger points in the gluteus minimus, medius, and maximus and piriformis due to point tenderness. DN was performed using 0.30 mm x 50 mm needles, which were left in place for 10 minutes. See Figure 1 for visual information on DN for the lateral hip. The physical therapist also instructed the patient to perform exercises to strengthen the hip abductors/extensors including side lying hip abduction, bridges, and exercise band-resisted side steps and applied passive hip flexor stretching with the patient positioned on a step while standing. At the final follow-up, the patient denied any pain at rest or with activity, had no functional limitations, and his Modified Oswestry Disability Index indicated only a mild level of disability (Table 1). In addition, he was able to return to golf. The physical therapist followed up with the patient four months later, and the patient remarked that DN was helpful, and that he would return as needed. 

Case 2

A 65-year-old overweight woman 10 years status post right THA with no other significant past medical history presented to the outpatient orthopedic physical therapy clinic in 2020 with a one-month history of 8/10 severity right posterior and anterior hip pain which began acutely after tripping, falling, and landing on her right hip. She reported that it felt like her “right hip wants to give out” and the pain was "sharp." She had visited her orthopedic surgeon and reportedly had no problems with her right hip THA, and no clinical or radiographic evidence of hip fracture, prosthesis loosening, failure, or infection. Patient initially had right THA due to significant osteoarthritis. Prior to her fall, she was not using an assistive device for ambulation. However, she was now using a walker due to increased ambulatory difficulty. Her Western Ontario and McMaster Universities Arthritis Index (WOMAC) was rated 65%. Patient was taking non-steroidal anti-inflammatory drugs to manage her symptoms. 

On observation, the physical therapist noted limitations in right hip passive range of motion and strength (Table 2), as well as the multiple trigger points in her lateral hip musculature (i.e., gluteus maximus, medius, and minimus, and piriformis). An examination of sensation and muscle stretch reflexes was normal. Considering the traumatic onset of symptoms, location of pain, limited mobility, and lack of an alternate cause of symptoms (e.g., THA or lumbar spine), the physical therapist considered a differential diagnosis of greater trochanteric pain syndrome and trochanteric bursitis.

**Table 2:** Initial and follow-up examination and outcome measures for Case 2 Abbreviations: numeric pain rating scale (NPRS), Western Ontario and McMaster Universities Arthritis Index (WOMAC) *Strength testing was scored out of a maximum of 5 (modified Medical Research Council manual muscle testing scale)

	Initial evaluation	Final follow-up
Passive hip range of motion (degrees)		
Flexion	55	95
Internal rotation	10	25
External rotation	30	50
Hip strength*		
Flexion	4-	4+
Abduction	4-	4+
Internal rotation	4	4+
External rotation	4	4+
Disability measures		
WOMAC raw score	63/96	16/96
WOMAC disability (%)	65	16
Pain (NPRS)	8/10	0/10

The physical therapist recommended a treatment plan (two visits per week over one month) to which the patient consented, and at each visit provided DN targeting gluteal trigger points and peri-trochanteric soft tissue. DN was performed using 0.30 mm x 50 mm needles, which were left in place for 10 minutes. The patient was instructed to perform exercises to strengthen hip extension and abduction including variations of side lying abduction, bridges, standing hip abduction and extension, and mini squats. At a final follow-up, the patient denied any pain, had improvements in her range of motion, strength, and WOMAC score (Table 2), and was not using any assistive device for ambulation, but was walking with a minimal limp. On follow-up one month after physical therapy discharge, the patient reported that her hip pain had returned to a moderate level (i.e., 4-6/10) but had not returned to its initial severity.

## Discussion

The current case series highlights the use of DN combined with conventional physical therapy to manage recurrent or new hip pain arising months or years after THA. Two elderly patients with a previous successful THA and clinical signs of a musculoskeletal source of recurrent hip pain exhibited improvements in hip strength, range of motion, and functional measures of disability after multiple sessions of DN, strengthening, and stretching. To our knowledge, this is the first manuscript to describe the use of DN in individuals with previous THA.

The current series may be compared to recent studies describing the use of DN in the post-surgical population. To be sure, the physiological mechanism behind pain relief from DN is complex and multi-faceted [5]. A case series of 14 patients found that DN led to significant improvements in pain, range of motion, and function in patients with chronic knee pain after TKA [6]. A case report described the improvement of hip pain and function with DN and strengthening and stretching exercises for a woman 13 weeks following arthroscopic labrum repair of the hip (without THA) [7]. Both studies reported no serious adverse events with DN in these patients, providing limited evidence that DN is safe when applied after lower extremity joint surgery. 

Limitations of this case series include a small sample size as well as a lack of detailed long-term follow-up. Future research should focus on outcomes of DN for THA with a longer follow-up, such as six months. Other limitations include limited generalizability as pain sources after THA may vary, different outcome assessments for each patient, lack of advanced imaging (i.e., magnetic resonance imaging, diagnostic ultrasound) to determine the exact cause of hip pain, compounding effects of time and non-steroidal anti-inflammatory drug use on symptom improvement, and unavailability of patient radiographs.

Further research is needed to examine the efficacy of DN for hip pain in individuals with previous THA. An ideal study would be a randomized controlled trial in which patients with muscular causes of hip pain at least one year after successful THA were recruited and randomized to either a standard physical therapy group or standard physical therapy plus DN group. Outcomes could include measures of pain, strength, range of motion, medication use, and patient-reported function/disability.

## Conclusions

Hip pain after THA is common yet has limited management options described in the literature. The current case series highlights improvement of hip pain using DN alongside strengthening and stretching exercises in two patients with previous THA. While the current cases suggest that DN may be an effective and safe treatment for such patients, additional research is needed to examine the efficacy of DN combined with conventional physical therapy in this patient population before it can be broadly recommended.
